# NLRX1 suppresses tumorigenesis and attenuates histiocytic sarcoma through the negative regulation of NF-λB signaling

**DOI:** 10.18632/oncotarget.8861

**Published:** 2016-04-20

**Authors:** Sheryl Coutermarsh-Ott, Alysha Simmons, Vittoria Capria, Tanya LeRoith, Justin E. Wilson, Bettina Heid, Casandra W. Philipson, Qizhi Qin, Raquel Hontecillas-Magarzo, Josep Bassaganya-Riera, Jenny P-Y Ting, Nikolaos Dervisis, Irving C. Allen

**Affiliations:** ^1^ Department of Biological Sciences and Pathobiology, Virginia Tech, VA-MD Regional College of Veterinary Medicine, Blacksburg, VA, USA; ^2^ Department of Genetics, Lineberger Comprehensive Cancer Center, University of North Carolina at Chapel Hill, Chapel Hill, NC, USA; ^3^ Virginia Tech, Virginia Bioinformatics Institute, Nutritional Immunology and Molecular Medicine Laboratory, Blacksburg, VA, USA; ^4^ Department of Small Animal Clinical Sciences, Virginia Tech, VA-MD Regional College of Veterinary Medicine, Blacksburg, VA, USA

**Keywords:** NLR, Nod-like receptor, urethane, cancer, inflammation

## Abstract

Histiocytic sarcoma is an uncommon malignancy in both humans and veterinary species. Research exploring the pathogenesis of this disease is scarce; thus, diagnostic and therapeutic options for patients are limited. Recent publications have suggested a role for the NLR, NLRX1, in acting as a tumor suppressor. Based on these prior findings, we hypothesized that NLRX1 would function to inhibit tumorigenesis and thus the development of histiocytic sarcoma. To test this, we utilized *Nlrx1^−/−^* mice and a model of urethane-induced tumorigenesis. *Nlrx1^−/−^* mice exposed to urethane developed splenic histiocytic sarcoma that was associated with significant up-regulation of the NF-λB signaling pathway. Additionally, development of these tumors was also significantly associated with the increased regulation of genes associated with AKT signaling, cell death and autophagy. Together, these data show that NLRX1 suppresses tumorigenesis and reveals new genetic pathways involved in the pathobiology of histiocytic sarcoma.

## INTRODUCTION

Histiocytic sarcoma is a rare, malignant neoplasm with a phenotypic profile consistent with an interstitial dendritic cell or macrophage origin. In human patients, this disease occurs most commonly in the intestinal tract, skin, soft tissue, and lymph nodes though case reports have also identified it in the central nervous system and stomach [1, 2]. Definitive diagnosis is often difficult due to its variable clinical presentation, as well as, its similarities to other histiocytic disorders such as hemophagocytic syndrome, malignant histiocytosis and monocytic leukemia. While still rare, this neoplasm is more common in veterinary medicine, where it is primarily a disease of dogs. Indeed, the bulk of studies of histiocytic sarcoma have arisen from cases in veterinary medicine. Treatment options in both human and veterinary medicine are limited and include complete surgical resection (when possible) coupled with chemotherapy or palliative radiation. However, in all species, treatment is often unsuccessful and the disease is typically fatal.

Pattern recognition receptors (PRRs) are important components of the innate immune system that are involved in the promotion and/or regulation of inflammation. These receptors recognize pathogen associated molecular patterns (PAMPs) and/or damage associated molecular patterns (DAMPs), which are products released by stressed or dying cells. PRRs that have been shown to significantly modulate the pathogenesis of neoplasia, include the Toll-like receptors (TLRs) and the NOD-like receptors (NLRs). TLRs are located on the surface of the cell, as well as, within endosomes and have been shown to play a role in lung cancer, breast cancer, and colon cancer [3-7]. NLRs are intracellular sensors and are well-studied in the context of colitis associated colorectal cancer [8, 9]. However, their involvement in other neoplasms is generally less defined and currently an area of intense research focus.

At least 34 NLR family members have been identified in mice and at least 22 in humans, the majority of which have yet to be functionally characterized [10]. Of the characterized NLRs, the majority appear to function as molecular effectors that form multi-protein complexes. For example, the most widely studied NLR family members form a multi-protein complex with the adaptor protein ASC and caspase-1, termed the inflammasome that is responsible for the production of the mature forms of the pro-inflammatory cytokines IL-1β and IL-18. However, a second sub-group of NLRs has recently been characterized that primarily function as non-inflammasome forming, regulatory NLRs. Members of this sub-group include NOD1 and NOD2, NLRX1 and NLRC3 (reviewed in [9]).

NLRX1 has been shown to be an important regulator of critical pathways associated with both inflammation and tumorigenesis. These include roles in the inhibition of NF-λB signaling, Type-I IFN production, and ROS production, as well as, the promotion of autophagy [11-15]. However, beyond these initial characterization studies, many questions remain unanswered regarding the function of this unique protein. The majority of studies investigating NLRX1 have focused on its role in host-pathogen interactions. However, the pathways modulated by NLRX1 are also typically dysregulated during tumorigenesis. Thus, we hypothesized that NLRX1 significantly inhibits tumorigenesis through regulating one or more of these previously characterized pathways. Here, we utilized *Nlrx1^−/−^* mice in a model of urethane induced tumorigenesis. Our data reveal that *Nlrx1^−/−^* mice are sensitive to urethane treatment and develop histiocytic sarcoma in the spleen that is associated with increased NF-λB signaling. We also identify a diverse range of genes associated with common cancer pathways, AKT signaling, cell death, and autophagy that are also significantly up-regulated in the *Nlrx1^−/−^* mice during histiocytic sarcoma. Collectively, our results further confirm that NLRX1 functions as a tumor suppressor and extends these findings to histiocytic sarcoma, which is an understudied cancer with few biomarkers.

## RESULTS

### NLRX1 is differentially regulated in multiple human cancers

**Figure 1:**
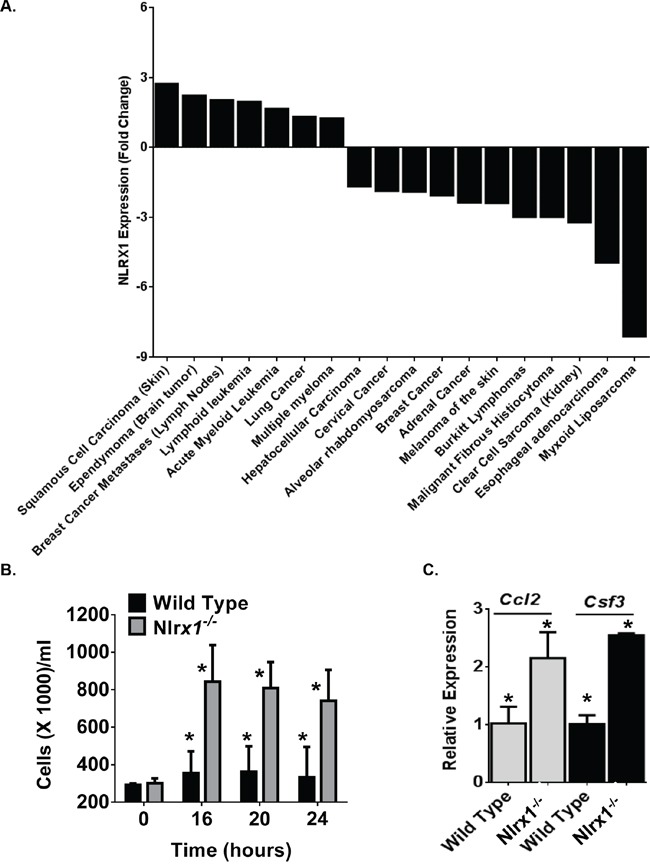
*NLRX1* Gene Expression is Significantly Dysregulated in Diverse Human Neoplasms **A.** A retrospective analysis of gene expression metadata from samples collected from human subjects revealed that *NLRX1* expression was significantly dysregulated in a diverse range of cancer subtypes. Data shown were determined to be significant changes in *NLRX1* expression between the tumor sample and either adjacent tissue from the same subject or a comparable tissue from an unaffected control subject based on the specific parameters established in each individual study. **B-C.** Macrophages from *Nlrx1^−/−^* mice rapidly proliferate and release increased cytokines associated with cell growth and migration. B) Bone marrow derived macrophages from *Nlrx1^−/−^* mice significantly expand under standard tissue culture conditions. C) Significant increases in *Ccl2* (Monocyte Chemotactic Protein-1) and *Csf3* (GCSF) gene expression were observed in *Nlrx1^−/−^* macrophages compared to wild type macrophages. Data are representative of 5 independent studies. *p<0.05.

To gain broader insight into the contribution of NLRX1 in cancer, we conducted a retrospective evaluation of publically available gene expression metadata compiled from 18 human studies (Figure 1A). Each study focused on a specific type or sub-type of cancer and evaluated gene expression levels between the tumor specimen and either adjacent healthy tissue or specimens from comparable tissue in unaffected subjects. The change in *NLRX1* expression was deemed significant based on the parameters of each individual study. Our data analysis revealed that *NLRX1* is differentially regulated in a diverse range of human cancers (Figure 1A). For example, at the extremes, *NLRX1* was found to be up-regulated 2.72 fold in squamous cell carcinoma of the skin compared to normal skin, while being down-regulated 8.1 fold in high grade myxoid liposarcoma tumors compared to normal adipose tissue (Figure 1A). While no human histiocytic sarcoma studies have been conducted, *NLRX1* gene expression data was evaluated for malignant fibrous histiocytoma (Figure 1A). Malignant fibrous histiocytoma, like histiocytic sarcoma, is controversial in origin though histiocytic cells are thought to be a major contributor. In both humans and canines, this is a soft tissue sarcoma and, in dogs, occurs most commonly in the spleen and skin. The fact that NLRX1 is downregulated in this neoplasm may suggest a similar pattern in human histiocytic sarcoma. Together, these data reveal that NLRX1 plays a complex role in tumorigenesis in humans and suggests that additional studies are needed to better define the contribution of this gene in patient populations.

### NLRX1 deficiency results in increased cell proliferation and chemokine production

The role of NLRX1 in the regulation of pathways associated with tumorigenesis is not well defined. A recent pair of studies have suggested that NLRX1 functions as a tumor suppressor through modulating apoptosis [16, 17]. In one study, NLRX1 expression was found to differentially regulate resistance to extrinsic and intrinsic apoptotic signals in transformed, but not primary murine embryonic fibroblasts [17]. In the other study, NLRX1 was found to function as a tumor suppressor by regulating TNF induced apoptosis in immortalized cell lines [16]. In the same study, NLRX1 overexpression was found to compromise clonogenicity, growth and migration [16]. To complement these prior studies, we sought to directly evaluate the contribution of NLRX1 on cell proliferation and growth. Bone marrow derived macrophages were harvested from wild type and *Nlrx1^−/−^* mice and allowed to differentiate for 5-7 days following standard protocols [18]. Cells were quantified and re-plated at 275,000 cells/ml in standard growth media without the addition of FBS (Figure 1B). Over the course of 24 hours, cells were counted using both trypan blue and a hemacytometer, as well as, propidium iodide (PI) staining and assessments with an automated cell counter (Figure 1B). Both live cells and dead cells were counted using these techniques. Under these conditions, wild type macrophages did not increase in number over the 24 hour time course. However, we did observe significant expansion of *Nlrx1^−/−^* macrophages over the 24 hour time course (Figure 1B). The *Nlrx1^−/−^* macrophages more than doubled the number of wild type macrophages 16 hours after re-plating (Figure 1B). Further assessments of gene expression revealed that chemokines associated with macrophage proliferation and recruitment, including *Ccl2* (MCP1) and *Csf3* (GCSF), were up-regulated in *Nlrx1^−/−^* macrophages compared to the wild type cells (Figure 1C). Unlike the prior studies, we did not observe differences in cell death. However, it should be noted that the prior studies induced apoptosis through TNF stimulation, glycolysis inhibition, increased cytosolic calcium flux, and endoplasmic reticulum stress [16, 17]. The current study evaluated cells with minimal stimulation beyond the overnight incubation in serum-free conditions. Thus, it is highly likely that the differences in cell death are associated with cell type, temporal, and stimulation specific mechanisms.

### NLRX1 attenuates disease pathogenesis following urethane exposure

**Figure 2:**
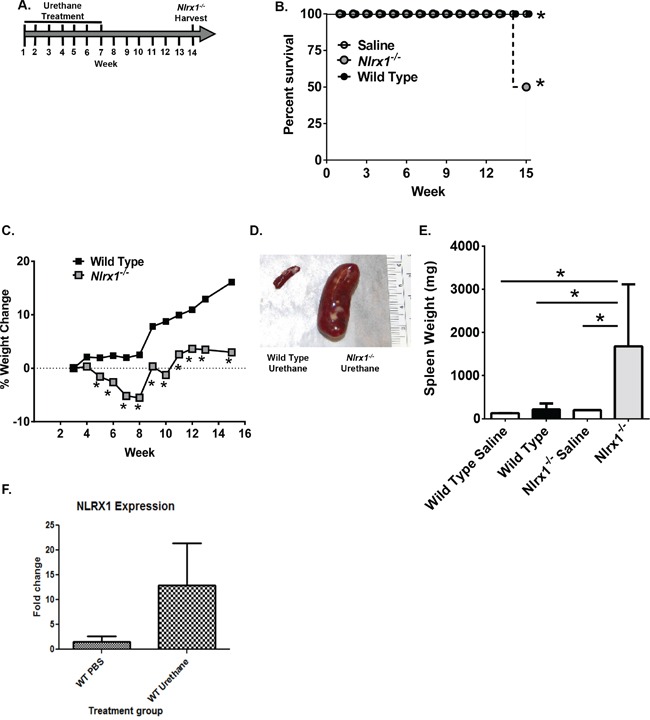
NLRX1 Improves Survival and Morbidity Following Urethane Exposure **A.** Schematic of the urethane model. *Nlrx1^−/−^* and wild type mice received 7 injections of 1g/kg body weight of urethane in saline over the course of 7 weeks. **B.** Kaplan-Meier plot of *Nlrx1^−/−^* and wild type mouse survival. **C.** *Nlrx1^−/−^* mice demonstrated a significant decrease in weight gain over the course of the model compared to the wild type animals. **D.** Necropsy revealed palpable masses in the peritoneal cavity associated with hypersplenomegaly in the *Nlrx1^−/−^* mice. **E.** Urethane treatment resulted in significant increases in spleen weight in the *Nlrx1^−/−^* mice compared to wild type animals. **F.** Urethane treatment increases *Nlrx1* expression in wild type mice. Data are representative of 3 independent studies. *Nlrx1^−/−^* urethane, n=10; wild type urethane, n=28; *Nlrx1^−/−^* saline, n=3; wild type saline, n=3. *p<0.05.

To better characterize the role of NLRX1 in cancer, we subjected *Nlrx1^−/−^* mice to a urethane (ethyl carbamate)-induced tumor model (Figure 2A). Urethane induced tumor formation is a prototypical and highly reproducible animal model of carcinogenesis that is traditionally utilized to study lung cancer [19–21]. Repeated urethane exposure consistently results in pulmonary adenoma and adenocarcinoma formation [21]. Similar to some human adenocarcinomas, urethane induces specific mutations in *Kras* at codon 61 and mutations in p53 during later stages of disease progression [20]. Wild type and *Nlrx1^−/−^* mice were administered weekly intraperitoneal injections of 1g/kg body weight of urethane diluted in saline for a total of 7 weeks (Figure 2A). Survival, weight loss, and clinical parameters associated with disease progression were routinely monitored. We evaluated disease progression and tumorigenesis at weeks 7 and 14. We did not observe any significant pathological changes at week 7, following the final urethane injection (Supplementary Figure 1). However, by week 14, 50% of the *Nlrx1^−/−^* mice had developed palpable masses in their peritoneal cavity and required euthanasia (Figure 2B). Additionally, the urethane treated *Nlrx1^−/−^* mice failed to thrive throughout the duration of the study and demonstrated significantly decreased weight gain compared with the wild type animals starting 5 weeks after the initial exposure to urethane (Figure 2C). At necropsy, the palpable masses originally detected in the *Nlrx1^−/−^* animals were identified as markedly enlarged spleens (Figure 2D). Spleens from all animals were weighed and those from the urethane treated *Nlrx1^−/−^* mice were indeed significantly larger than all of the other genotypes and treatments (Figure 2E). No gross lesions were identified in any additional tissues or organs. Following urethane treatment, *Nlrx1* expression was increased in wild type animals that were resistant to histiocytic sarcoma (Figure 2F).

### NLRX1 suppresses the development of histiocytic sarcoma

**Figure 3:**
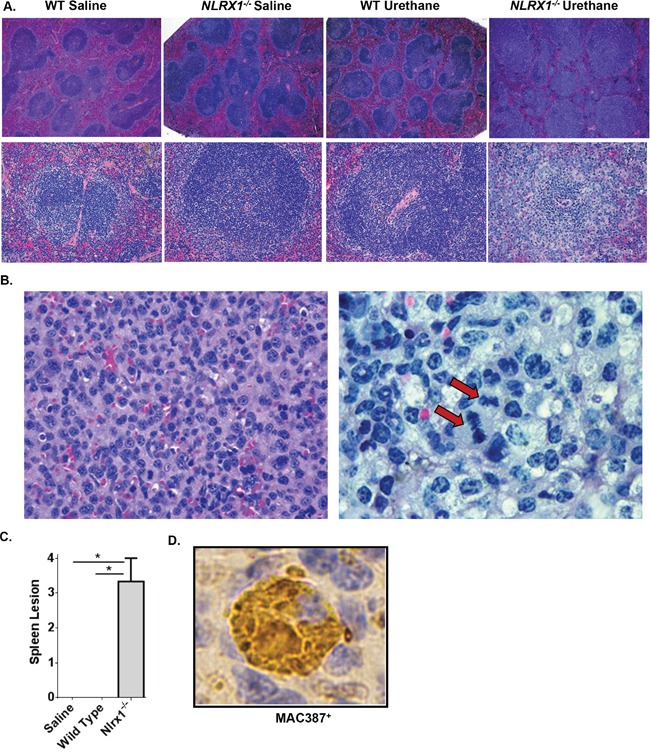
NLRX1 Attenuates the Development and Progression of Histiocytic Sarcoma Evaluation of histopathology revealed that urethane treatment resulted in the development of histiocytic sarcoma in *Nlrx1^−/−^* mice. **A.** Evaluation of H&E stained sections revealed a significant expansion of the white pulp and increased monocyte populations in spleens from urethane treated *Nlrx1^−/−^* mice compared to the saline treated and urethane treated wild type animals. **B.** Higher magnification evaluation revealed that all of the lesions contained high-grade malignancies consisting of markedly pleomorphic monocytes containing multiple mitotic figures (arrow). **C.** Semi-quantitative scoring of histopathology revealed a significant increase in spleen lesions in the *Nlrx1^−/−^* mice compared to the wild type animals following urethane treatment. **D.** Immunohistochemistry using MAC387 (Abcam) was utilized to better classify the pre-dominate cell populations present in the spleen. All of the monocytes associated with spleen lesions were strongly positive for MAC387, suggesting that they are predominately macrophages. Data are representative of 3 independent studies. *Nlrx1^−/−^* urethane, n=10; wild type urethane, n=28; *Nlrx1^−/−^* saline, n=3; wild type saline, n=3. *p<0.05.

The increased urethane sensitivity observed in the *Nlrx1^−/−^* mice was directly correlated with splenomegaly. Subsequent histopathological assessments by two board certified veterinary pathologists (T.L.R. and S.C.O.) revealed that spleens from the urethane treated *Nlrx1^−/−^* mice were characterized by a significant expansion of the white pulp by high numbers of pleomorphic, mononuclear cells with frequent mitotic figures, thus consistent with a diagnosis of histiocytic sarcoma (Figure 3A-3B). No significant pathology was observed in the spleens from the saline treated wild type and *Nlrx1^−/−^* mice or the urethane treated wild type animals (Figure 3A). A semi-quantitative assessment of spleen histopathology, based on number of lesions and percent area affected (scored on a scale of 0 – 3), revealed a significant increase in the spleen lesion score for the urethane treated *Nlrx1^−/−^* mice compared to all other genotypes and treatments (Figure 3C). Additional flow cytometry assessments of spleen cellularity, conducted at the time of harvest, confirmed the increased numbers of monocyte derived cells in the *Nlrx1^−/−^* spleens compared to the wild type. T-cell, B-cell, and NK-cell populations were not significantly different between wild type and *Nlrx1^−/−^* spleens (data not shown). Immunohistochemistry for MAC387 was performed and neoplastic cells showed strong cytoplasmic positive staining (Figure 3D). Based on these results, our data suggests that macrophages are the dominate cell population associated with histiocytic sarcoma in the *Nlrx1^−/−^* mice. Together, these data suggest that NLRX1 functions to attenuate histiocytic sarcoma induced by urethane and further support a role for this unique NLR in tumor suppression.

### NLRX1 attenuates urethane induced tumorigenesis in the lung and inflammation in the liver

**Figure 4:**
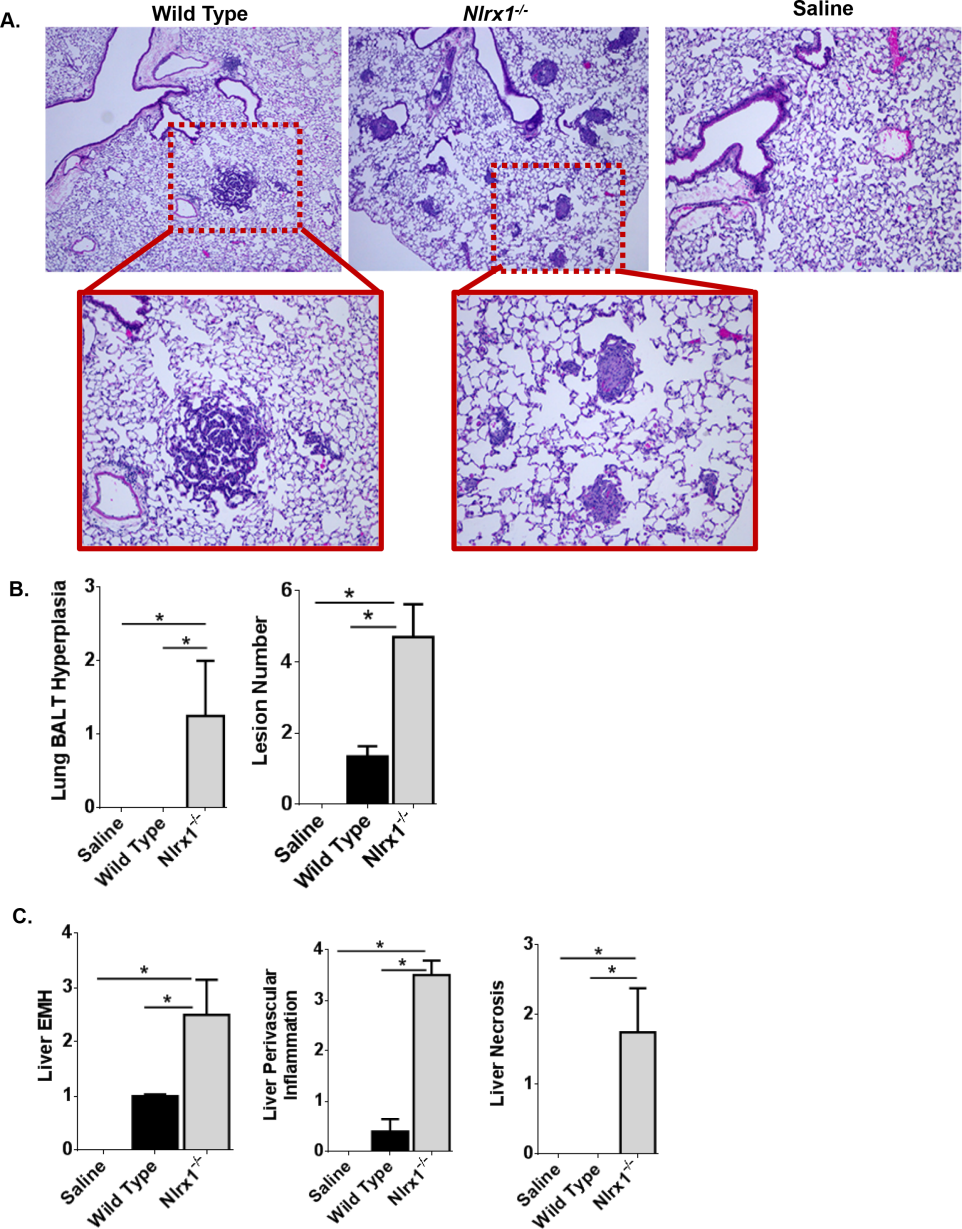
NLRX1 Attenuates Urethane Induced Lesions in the Lung and Liver **A.** Evaluation of histopathology revealed that urethane exposure increased lung lesions in both *Nlrx1^−/−^* and wild type mice. **B.** Semi-quantitative scoring revealed a significant increase in bronchial associated lymphoid tissue (BALT) hyperplasia and the number of lesions in lungs harvested from urethane treated *Nlrx1^−/−^* mice. **C.** In addition to the lungs, urethane exposure also resulted in increased liver lesions in the wild type and *Nlrx1^−/−^* mice. *Nlrx1^−/−^* mice developed significant increases in extramedullary hematopoiesis, perivascular inflammation, and necrosis compared to wild type animals. Data are representative of 3 independent studies. *Nlrx1^−/−^* urethane, n=10; wild type urethane, n=28; *Nlrx1^−/−^* saline, n=3; wild type saline, n=3. *p<0.05.

Typically, lung cancer progression is much slower in the urethane model, especially in C57Bl/6 mice and pathology is usually evaluated at least 24 weeks following the initial exposure. However, due to the sensitivity of the *Nlrx1^−/−^* mice to urethane and the rapid development of histiocytic sarcoma in the spleen, we evaluated lung histopathology 14 weeks following the initial exposure to urethane. In the wild type mice, we observed a small number of lesions in each treated animals consistent with urethane exposure (Figure 4A). Similar lesions were also observed in the *Nlrx1^−/−^* mice (Figure 4A). However, in the urethane treated *Nlrx1^−/−^* animals, we observed significantly higher numbers of tumor lesions (mean = 4.7 lesions) compared to the wild type animals (mean = 1.3) as well as a significant increase in bronchial associated lymphoid tissue (BALT) not seen in the wild type animals (Figure 4B). Thus, even at this earlier time-point in the urethane model, the wild type animals had begun forming the characteristic lung lesions associated with urethane exposure and, consistent with the morbidity data and findings in the spleen, the *Nlrx1^−/−^* mice were more sensitive. At necropsy, we also collected the liver for microscopic evaluation. Histopathologic assessments revealed that urethane exposure increased liver extramedullary hematopoiesis (EMH) and perivascular inflammation in all of the treated animals when compared to untreated animals (Figure 4C). Moreover, we observed a significant increase in both parameters in the *Nlrx1^−/−^* mice compared to the wild type animals. In addition to the increased EMH and inflammation, we also observed a significant increase in liver necrosis in the urethane treated *Nlrx1^−/−^* mice (Figure 4C). This necrosis was not observed in the wild type animals. The increased mass formation in the lung and increased inflammation in the lung and liver observed in the urethane treated *Nlrx1^−/−^* mice is consistent with previous reports that identify NLRX1 as a negative regulator of inflammatory signaling pathways [22]. Likewise, the increased necrosis in the liver is consistent with previously reported findings in other models that suggest a role for NLRX1 in the modulation of cell death, autophagy, and cell metabolism [12, 16].

### Genes associated with cancer, cell death and autophagy are significantly up-regulated in histiocytic sarcoma in *Nlrx1^−/−^ mice*

NLRX1 has been previously shown to modulate inflammatory signaling pathways, cell death, autophagy, and reactive oxygen species (ROS) production [8, 16]. To better address the signaling pathways that are dysregulated in the *Nlrx1^−/−^* mice during tumorigenesis, we profiled gene expression in the spleen following urethane exposure. Spleens were harvested from both urethane treated and untreated wild type and *Nlrx1^−/−^* animals and total RNA was extracted (Supplementary Figure 2). The RNA from 3-5 randomly chosen spleens from each genotype and treatment were pooled in equal amounts and cDNA was generated (Supplementary Figure 2). Different random pools of RNA were evaluated on each Superarray. The expression of 241 genes was evaluated using a panel of Superarrays (Qiagen) chosen to evaluate pathways associated with cancer, inflammation, cell death, and autophagy using methods previously described by the authors [14]. Gene expression was determined following the manufacturer's protocols, which are based on the ΔΔCt method. The expression of each gene on the array was first normalized to a panel of 8 housekeeping genes and the change in gene expression between the respective urethane treated versus untreated wild type and *Nlrx1^−/−^* spleens was determined. The change in gene expression between the wild type and *Nlrx1^−/−^* spleens was then calculated and displayed as the fold change for each gene on the various arrays.

**Figure 5:**
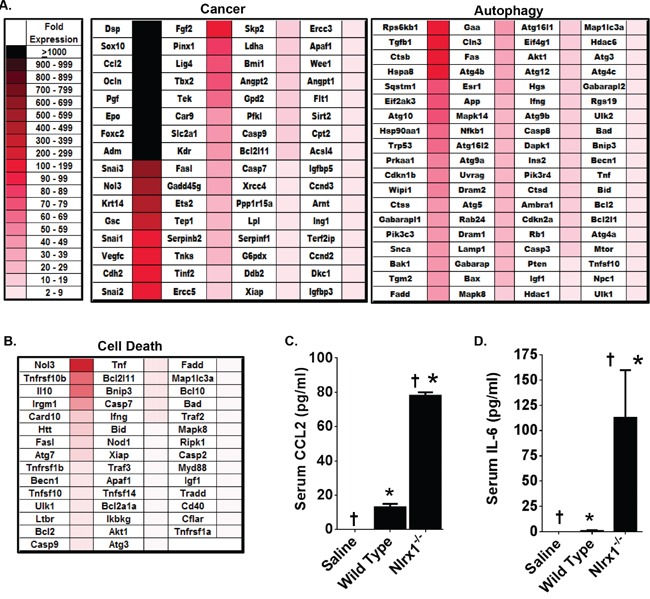
Genes Associated with Cancer, Autophagy, and Cell Death Are Significantly Up-Regulated in NLRX1 Deficient Mice during Histiocytic Sarcoma Gene transcription was profiled from RNA collected from wild type and *Nlrx1^−/−^* spleens 14 weeks following the initial saline or urethane exposure. **A-B.** Heatmap reflecting the change in gene expression of all genes associated with common cancer pathways, autophagy, and cell death that were identified as being significantly up-regulated in the spleen following urethane treatment of *Nlrx1^−/−^* mice compared to the urethane treated wild type animals. Analysis was based on the ΔΔCt method, where all data was standardized to the average gene expression for a panel of 8 housekeeping genes and normalized to the respective untreated *Nlrx1^−/−^* and untreated wild type spleens. Greater than a 2-fold change in gene expression was considered significant. Three - five randomly selected spleens from each genotype and treatment group were selected and pooled for profiling studies. **C-D.** A significant increase in serum IL-6 and CCL2 levels were detected in the *Nlrx1^−/−^* mice compared to wild type animals 14 weeks after the initial urethane injection. Cytokine levels were determined by ELISA. *Nlrx1^−/−^* urethane, n=6; wild type urethane, n=10; *Nlrx1^−/−^* saline, n=3; wild type saline, n=3. *p<0.05.

Because it is such a rare disease, very little data exists regarding gene expression or biochemical signaling pathways that are dysregulated in histiocytic sarcoma. Thus, we initially sought to evaluate genes commonly associated with cancer signaling. This initial evaluation revealed that 64 genes associated with tumorigenesis were significantly up-regulated in *Nlrx1^−/−^* spleens with histiocytic sarcoma compared to the wild type (Figure 5A). The genes with the greatest differences in expression (>1000 fold increase) included: *Dsp*, *Sox10*, *Ccl2*, *Ocln*, *Pgf*, *Epo*, *Foxc2*, and *Adm* (Figure 5A). Interestingly, all of these genes, with the exception of *Foxc2*, have been previously associated with various types of sarcoma in either human or rodent studies and increased *CCL2* gene expression has been directly correlated with histiocytic sarcoma in canine patients [23]. In addition to increased expression of cancer associated genes, we also observed increased expression of 76 genes associated with autophagy (Figure 5A). The genes with the greatest differences in expression (100-199 fold increase) included: *Rps6kb1*, *Tgfb1*, *Ctsb*, and *Hspa8* (Figure 5A). NLRX1 has been previously shown to positively regulate autophagy following virus exposure [13]. However, in the current study, NLRX1 deficiency was found to result in the elevation of several autophagy genes. This may suggest that under the conditions evaluated in this study, NLRX1 acts as a negative regulator of autophagy. However, it is important to note that changes in gene expression do not necessarily correlate to changes in protein expression. Therefore, additional studies into protein expression would be necessary to better elucidate the effects of NLRX1 on autophagy in HS. We also observed increased expression of 44 genes associated with cell death (Figure 5B). These genes were further stratified based on general roles in either necrosis or apoptosis (Supplementary Figure 3). Together, these data suggest that NLRX1 negatively regulates gene expression associated with autophagy, and more broadly cell death, during tumorigenesis. This finding is consistent with a previous study that found NLRX1 functions as a tumor suppressor through the regulation of TNF induced apoptosis [16].

Significant increases in serum CCL2 levels have been found in canine cases of disseminated histiocytic sarcoma [23]. Likewise, *Ccl2* appears to be up-regulated in macrophages from *Nlrx1^−/−^* mice and was identified as being one of the genes with the greatest level of up-regulation in the spleen during histiocytic sarcoma (Figures 1C and 5A). Thus, we evaluated serum protein levels of CCL2 by ELISA (Figure 5B). Consistent with the increased expression of *Ccl2* in the spleen, we observed a significant increase in serum CCL2 levels following urethane treatment in *Nlrx1^−/−^* mice compared to the wild type animals (Figure 5B). Prior studies have also evaluated other pro-inflammatory cytokines in the context of histiocytic sarcoma, including IL-6 [23]. While these studies did not find any correlation between IL-6 and tumor progression, this cytokine has been reported to be increased in the absence of NLRX1 [14]. Thus, we also evaluated serum protein levels of IL-6 by ELISA and found that the levels of this cytokine were significantly increased in urethane treated *Nlrx1^−/−^* mice compared to the wild type animals (Figure 5C). Together with the gene expression findings, these data show that NLRX1 attenuates inflammation and tumorigenesis through the negative regulation of genes associated with cancer, autophagy, and cell death during histiocytic sarcoma.

### NLRX1 negatively regulates NF-λB and AKT signaling in histiocytic sarcoma

**Figure 6:**
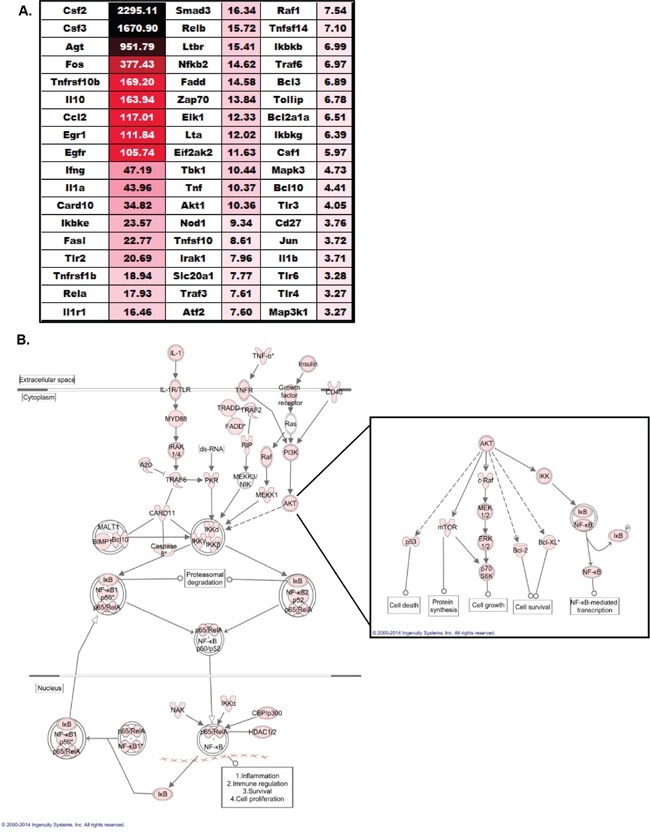
NLRX1 Negatively Regulates NF-λB and AKT Signaling during Tumorigenesis **A.** Heatmap and fold change in expression of all genes associated with NF-λB signaling that were identified as being significantly up-regulated in the spleen following urethane treatment of *Nlrx1^−/−^* mice compared to the urethane treated wild type animals. Analysis was based on the ΔΔCt method, where all data was standardized to the average gene expression for a panel of 8 housekeeping genes and normalized to the respective untreated *Nlrx1^−/−^* and untreated wild type spleens. Greater than a 2-fold change in gene expression was considered significant. Three – five randomly selected spleens from each genotype and treatment group were selected and pooled for profiling studies. **B.** Evaluation of all gene expression data using Ingenuity Pathway Analysis revealed a significant increase in NF-λB and AKT signaling in *Nlrx1^−/−^* spleens following urethane exposure compared to wild type. Pink icons represents genes that were significantly up-regulated (>2 fold change in expression) in the *Nlrx1^−/−^* spleens compared to the change in expression observed in the wild type spleens following urethane treatment. *Nlrx1^−/−^* urethane, n=5; wild type urethane, n=5; *Nlrx1^−/−^* saline, n=5; wild type saline, n=5.

In general, the genes and pathways that were found up-regulated were highly diverse and covered a broad spectrum of pathways associated with tumorigenesis. This suggests that NLRX1 likely indirectly regulates these pathways through its effects on one or more essential regulatory pathways up-stream from the genes evaluated. Prior studies have shown that NLRX1 negatively regulates the type-I interferon response and the NF-λB signaling cascade following virus exposure [14, 15]. While no prior data has associated IFN signaling with the urethane model, several lines of evidence indicate that the urethane model is potentiated by inflammation associated with increases in NF-λB signaling [19, 24]. Evaluation of the gene expression data did not reveal any significant differences in expression among genes generally associated with IFN signaling between the wild type and *Nlrx1^−/−^* spleens. Further analysis of pathways that were enriched in the spleen following urethane treatment also did not identify the IFN signaling pathway as being significantly dysregulated (data not shown). Thus, IFN signaling does not appear to play a role in either histiocytic sarcoma or NLRX1 function in this model. However, unlike the findings of IFN signaling, we did observe significant differences in genes associated with the NF-λB signaling cascade (Figure 6A). Our analysis revealed that 54 genes associated with NF-λB signaling were significantly up-regulated (> 2-fold increase in expression) in the spleens from urethane treated *Nlrx1^−/−^* mice compared to the wild type animals (Figure 6A). Two genes, *Csf2* and *Csf3*, were found to have the highest fold change in expression (>1000 fold) (Figure 6A). Seven additional genes had greater than 100-fold changes in expression between the *Nlrx1^−/−^* and wild type spleens, including *Agt*, *Fos*, *Tnfrsf10b*, *Il10*, *Ccl2*, *Egr1*, and *Egfr* (Figure 6A). All expression data was analyzed using Ingenuity Pathway Analysis (IPA) to identify relationships and pathways that were enriched in the urethane treated *Nlrx1^−/−^* spleens compared to the wild type spleens. The IPA analysis confirmed that the NF-λB signaling pathway was significantly up-regulated during histiocytic sarcoma in the *Nlrx1^−/−^* mice (Figure 6B). These data are consistent with prior studies supporting a role for NLRX1 in the negative regulation of NF-λB [14]. The IPA analysis also revealed a significant increase in TNF signaling (Figure 6B), which is consistent with the recent study that suggested NLRX1 functions as a tumor suppressor through modulating this pathway in cancer cells [16]. Our IPA analysis further revealed a significant increase in the AKT signaling pathway (Figure 6B). This was unexpected, as no prior studies have shown an association between NLRX1 function and AKT signaling. Each pathway downstream of AKT signaling was found to be up-regulated in the absence of NLRX1 during histiocytic sarcoma (Figure 6B). While more studies are clearly necessary, these findings suggest that NLRX1 may also function, either directly or indirectly, to negatively regulate AKT signaling during cancer. Together, these data suggest that the sensitivity of the *Nlrx1^−/−^* mice to urethane is at least in part associated with the up-regulation of gene transcription associated with increased NF-λB and AKT signaling.

## DISCUSSION

Traditionally, the roles of NLRX1 have predominately been studied in the context of host-pathogen interactions. However, recent studies have expanded the function of this unique NLR to include roles in the regulation of metabolism, cell death, and cancer [12, 16, 17]. The results obtained in the present study support previous findings and strongly suggest that NLRX1 functions to attenuate tumor progression. Mechanistically, our data suggest that this is through the downregulation of NF-λB signaling, as this pathway is significantly up-regulated in the *Nlrx1^−/−^* mice following urethane exposure. Thus, in the current study, we have identified NLRX1 as an important tumor suppressor and characterized a group of genes downstream of NF-λB signaling that are significantly up-regulated in the *Nlrx1^−/−^* animals that contribute to the development of histiocytic sarcoma.

In addition to our current findings, prior studies have also evaluated NLRX1 in cancer utilizing either xenograft models or models of inflammation driven colorectal cancer [16, 17, 25, 26]. In the xenograft study, nude mice were injected with RKO colon carcinoma cells, where NLRX1 was either stably knocked down or overexpressed [16]. In the presence of TNF, NLRX1 knockdown resulted in significantly increased tumor volume, whereas overexpression attenuated tumor growth [16]. To evaluate the role of NLRX1 in CAC, tumorigenesis was induced in wild type and *Nlrx1^−/−^* mice by treating the animals with the chemical carcinogen azoxymethane (AOM) immediately prior to dextran sulfate sodium (DSS) exposure [17, 25, 26]. In this model, inflammation associated with DSS exposure functions as a tumor promoter. The results show that *Nlrx1^−/−^* mice were significantly more sensitive to inflammation driven tumorigenesis compared to similarly treated wild type animals [17, 25, 26]. Significantly higher numbers and larger-sized polyps were observed in the *Nlrx1^−/−^* mice [17]. The *Nlrx1^−/−^* mice were also found to have increased colon inflammation associated with DSS exposure [17]. In both the xenograft as well as the AOM/DSS models, the loss of NLRX1 was suggested to have implications in cell death leading to tumorigenesis, which is supported by our data as well. Findings from the xenograft study suggest that NLRX1 appears to sensitize the cells to TNF induced cell death through a Caspase-8 dependent mechanism and maintains ATP levels through the regulation of mitochondrial Complex I and Complex III activities [16]. A similar mechanism is described in the colitis associated cancer (CAC) studies [17]. In the CAC studies, *in vivo* tumorigenesis data was supported by correlations to murine embryonic fibroblast data that suggested NLRX1 expression mediates resistance to extrinsic apoptotic signals, while also conferring susceptibility to intrinsic apoptotic signals [17].

The results of the current study support a role for NLRX1 in mediating cell death under neoplastic conditions, but not in primary macrophages. Our study identified 44 genes associated with cell death that were upregulated in the spleens of *Nlrx1^−/−^* mice with histiocytic sarcoma. Interestingly, the majority of cell death genes upregulated in these mice were related to apoptosis; however, no clear link to either intrinsic or extrinsic apoptosis was identified. It is also unclear if the up-regulation of genes associated with cell death was directly associated with the neoplastic macrophages or associated with the general tissue damage that occurred in the spleen during histiocytic sarcoma progression. To expand upon these findings, we conducted basic assessments of cell death associated with our proliferation studies using primary bone marrow derived macrophages. Under our experimental conditions, we did not observe a significant difference in cell death between the wild type and *Nlrx1^−/−^* macrophages. Rather, we noted a significant increase in *Nlrx1^−/−^* cell proliferation. It is certainly possible that the increase in cell numbers that we observed could also be correlated with reduced cell death. In fact, increased cell proliferation and reduced cell death are commonly observed when essential pathways associated with tumorigenesis are disrupted [27]. The increased *Nlrx1^−/−^* macrophage proliferation is consistent with the increased splenic macrophage proliferation observed by histopathology in the spleens during histiocytic sarcoma. Likewise, the increase in expression of genes, such as *Ccl2* and *Csf3*, which are NF-λB regulated cytokines associated with macrophage proliferation and recruitment, are also consistent with our *in vivo* findings. Thus, the role of NLRX1 in regulating cell death (and proliferation) in normal versus neoplastic cells warrants further study.

In the present study, we sought to extend the assessments of NLRX1 and evaluate additional pathways modulated by this unique NLR in the context of tumorigenesis. Prior studies have shown that NLRX1 is a member of the regulatory NLR sub-group and functions to inhibit inflammatory signaling cascades, including IFN and NF-λB signaling (reviewed in [8]). While we did not observe any role for dysregulated IFN signaling in the development of histiocytic sarcoma in the *Nlrx1^−/−^* mice, we did find a strong correlation between tumorigenesis and the up-regulation of genes associated with NF-λB signaling. Prior mechanistic studies have shown that NLRX1 interacts with TRAF6 to negatively regulate NF-λB signaling [14]. The NF-λB signaling pathway directly regulates a wide range of biological functions beyond inflammation and is associated with almost every hallmark of cancer [27]. In the context of histiocytic sarcoma, there is a paucity of data pertaining to NF-λB signaling. Beyond a few incidental observations, no detailed studies have comprehensively evaluated this signaling pathway in histiocytic sarcoma. However, NF-λB signaling has been evaluated in other types of sarcoma. For example, in human hemangioma and angiosarcoma lesions, high levels of RelA and strong activation of the NF-λB/IL-6/STAT3 signaling axis has been previously reported [28]. These human data were complemented by studies using *Ink4a/Arf* deficient mice, which recapitulate genetic traits observed in human angiosarcoma patients and xenograft mice. In these models, animals developed angiosarcoma in the lung, liver, and spleen and systemic inhibition of Ikkβ, IL-6 or STAT3 significantly inhibited angiosarcoma growth [28]. In other sarcomas, inhibition of the NF-λB pathway has also been shown to significantly attenuate tumorigenesis. In a study utilizing a myxoid liposarcoma cell line, inhibition of the NF-λB signaling pathway was shown to decrease cell viability, reduce phosphorylation of NF-λB proteins, and attenuate caspase-3 regulated apoptosis [29]. Interestingly, the human metadata analysis described in the current study found the greatest decrease in NLRX1 expression in myxoid liposarcoma (Figure 1). This suggests a possible link between reduced NLRX1 levels and increased NF-λB signaling in this neoplasm.

In addition to up-regulation of the NF-λB cascade, we also observed increased AKT signaling during histiocytic sarcoma in the *Nlrx1^−/−^* mice. This result was quite unexpected as no prior studies have associated NLRX1 with AKT signaling. However a related protein, AIM2, has recently been shown to attenuate the progression of colon tumorigenesis, in part, through reducing AKT activation [30]. In this study, *Aim2^−/−^* mice were found to be highly sensitive to colitis associated colorectal cancer. The increased tumor burden in these mice was significantly attenuated in those animals treated with an AKT inhibitor, suggesting that up-regulation of this pathway is associated with tumorigenesis [30]. Unlike the NF-λB pathway, the contribution of AKT signaling in histiocytic sarcoma has been previously reported [31]. In human specimens, immunohistochemistry revealed high levels of p-AKT expression in 9 of 10 histiocytic sarcoma samples [31]. These human data were further supported using a novel *Pten^+/−^*Ink4a/Arf*^−/−^* double mutant mouse model of histiocytic sarcoma. In this model, loss of PTEN results in aberrant activation of the PI3K/AKT signaling pathway, as well as, the RAS/MAPK pathway [31]. Ultimately, this results in increased AKT, ERK1 and ERK2 phosphorylation and the eventual development of histiocytic sarcoma [31]. Interestingly, lymphomas arising in the *Ink4a/Arf^−/−^* mice did not show this increase in AKT signaling compared with normal tissue from wild type animals, suggesting that increased AKT signaling is specific to histiocytic sarcoma in this model [31].

We identified 194 genes up-regulated in the *Nlrx1^−/−^* mice following urethane administration that were associated with cancer signaling, autophagy and NF-λB signaling. Many interesting targets were identified that have not been previously associated with either histiocytic sarcoma or NLRX1 function. Of the individual genes and proteins that were found to be significantly dysregulated, IL-6 and CCL2 are highly interesting. We observed increased systemic levels of IL-6 in the serum of the *Nlrx1^−/−^* mice (Figure 5). In canine patients, previous reports have observed significant increases in IL-6 in metastatic splenic hemophagocytic histiocytic sarcoma [32]. Likewise, as mentioned above in human patients and mouse models, prior studies evaluating the NF-λB/IL-6/STAT3 axis in other types of sarcoma have reported increased IL-6 signaling and attenuation of tumorigenesis following treatment with IL-6 inhibitors [28]. Together, these findings suggest a major role for IL-6 in disease pathogenesis. Increased IL-6 levels have been previously reported in the *Nlrx1^−/−^* mice in other models associated with pathogen infection [14]. The direct association between NLRX1 and IL-6 signaling is presumed to result from increased NF-λB signaling found in the absence of NLRX1. A recent paper showed that enhanced IL-6 in *Nlrx1^−/−^* mice is consequential, as anti-IL6R therapy completely reduced colon polyps in these animals [25]. However, it is also important to note that increased IL-6 could also be an indirect response to the animal's clinical decline, rather than a direct effect of NLRX1 function on tumorigenesis. Additional mechanistic insight will be necessary to better define the relationship between NLRX1, IL-6 and histiocytic sarcoma pathogenesis. The increase in CCL2 (MCP-1), both *ex vivo* and *in vitro* is also an intriguing observation. Transcription of *CCL2* is highly regulated by NF-λB and the cytokine displays potent monocyte chemotactic activity. Increased CCL2 has been previously reported in veterinary patients with histiocytic sarcoma [23]. In this prior study, serum levels of CCL2 were significantly increased in dogs diagnosed with disseminated histiocytic sarcoma compared to healthy control animals [23]. While we focused on IL-6 and CCL2, many of the other genes identified are also highly interesting and should provide significant insight related to histiocytic sarcoma pathogenesis.

NLRX1 is a novel member of a unique regulatory sub-group of NLR family members that functions to negatively regulate diverse biochemical signaling cascades. Members of this regulatory sub-group function to control aberrant inflammation and other biologically relevant pathways associated with a variety of human diseases, including cancer. Here, we have shown that NLRX1 functions to suppress tumorigenesis, in part, through the negative regulation of NF-λB signaling. These findings are consistent with previous studies that have characterized NLRX1 as a tumor suppressor in other cancer models. We anticipate that future studies will better define the role of NLRX1 in cancer. Additionally, we have uncovered an extensive number of genes and pathways that have not previously been associated with histiocytic sarcoma. Thus, further studies of these pathways and dysregulated genes should identify novel targets for future therapeutic strategies to attenuate histiocytic sarcoma progression.

## MATERIALS AND METHODS

### Experimental animals

The generation and characterization of *Nlrx1^−/−^* mice have been previously described [14]. All experiments were conducted with 6 - 22 week old C57Bl/6 female mice. All animals were maintained under SPF conditions and received 5010 chow (LabDiet) and water *ad libitum*. All experiments were conducted in accordance with the NIH Guide for the Care and Use of Laboratory Animals and were conducted under institutional IACUC approval.

### Bone marrow derived macrophage studies

Bone marrow derived macrophages (BMDMs) were isolated from the femurs of C57Bl/6 and *Nlrx1^−/−^* mice using standard procedures [18]. The cells were cultured in Dulbecco's modified Eagle's medium (DMEM) with 10% fetal bovine serum (FBS), 20% L929-conditioned cell culture supernatant, 1x L-glutamine, and 1x non-essential amino acids for 5-7 days. BMDMs were sub-cultured with or without serum overnight. Both live and dead cells were counted at designated intervals using either trypan blue and a hemacytometer or PI staining and quantification with an automated cell counter (Cellometer Vision from Nexcelom Bioscience) following the manufacturer's protocols. A minimum of 1000 cells for each genotype and treatment were counted using the automated system. Supernatants were removed for cytokine measurements and total RNA was collected for gene expression analysis.

### Induction and assessment of histiocytic sarcoma

To assess the pathobiological effects of urethane, all mice were subjected to once weekly, i.p. injections of urethane (Sigma) at 1 g/kg diluted in 1X PBS for a total of 7 weeks [19]. Body weight, physical condition, and behavior were assessed at least 3 days per week throughout the course of each study. Mice were harvested at 7 or 14 weeks post-urethane exposure or when moribund. On the day of harvest, mice were euthanized via CO_2_ asphyxiation and approximately 1 ml of blood was collected by cardiac puncture. The lung, liver, and spleen were harvested from each animal. Portions of each tissue were placed either in formalin for histopathology or frozen and stored in the −80 for RNA/protein analysis. Samples of spleen were also prepared for flow cytometry.

### Histopathologic examination

Formalin-fixed tissues were routinely processed for histopathology. The paraffin-embedded tissues were sectioned at 5 μm and prepared for hematoxylin and eosin (H&E) staining. H&E stained sections were evaluated and scored by a board-certified veterinary pathologist (T.L.R and S.C.O) while blinded to genotype and treatment. Immunohistochemistry for MAC387 (Abcam) was used to evaluate monocyte populations in the spleen.

### Expression profiling

Total RNA was harvested from spleens following mechanical homogenization, lysis and RNA extraction using a FastRNA Pro Green Kit and the manufacturer's protocols (MP Biomedicals). The purified RNA was quantified and 1μg of RNA was pooled from 3-5 individual mice prior to the cDNA reaction, for analysis using the RT2 Profiler PCR Array Platform (SABiosciences). Samples were evaluated using the manufacturer's protocols for the following arrays: PAMM-033Z; PAMM-084Z; PAMM-011Z; PAMM-064Z; PAMM-025Z. Ingenuity Pathways Analysis (IPA) software was utilized to evaluate the array data. In addition to the profiling studies, RNA samples (5 μg) were also individually archived using a cDNA Archive Kit (ABI) and specifically targeted transcription products were quantified by real time PCR using commercially available primer/probe sets (ABI). All experimental samples were evaluated in triplicate and the relative expression was determined utilizing the ΔΔCt method by normalizing samples to the expression of the 18s rRNA housekeeping gene.

### Human metadata analysis

Human *NLRX1* expression was evaluated using a publically accessible microarray meta-analysis search engine (http://www.nextbio.com/b/search/ba.nb), as previously described [33]. The following array data series were analyzed to generate the human patient data: GSE7553; GSE66354; GSE32490; GSE37470; GSE34823; GSE7339; GSE36474; GSE50579; GSE9750; GSE28511; GSE29431; GSE19750; GSE7553; GSE29491; GSE2719; GSE49972; GSE13898; and GSE36982.

### Statistical analysis

Data are presented as the mean +/− standard error of the mean (SEM). Analysis Of Variance (ANOVA) followed by either Tukey-Kramer HSD or Newman-Keuls Post Test for multiple comparisons was performed on complex data sets for both individual experiments and composite data. Statistical significance for single data points were assessed by the Student's two-tailed t-test. Survival curves were generated utilizing the product limit method of Kaplan and Meier and comparisons were made using the log rank test. In all cases, a p-value of less than 0.05 was considered statistically significant.

## SUPPLEMENTARY FIGURES


