# Supplementary material for: NLRX1 suppresses tumorigenesis and attenuates histiocytic sarcoma through the negative regulation of NF-λB signaling
Source: Oncotarget. 2016 Apr 20;7(22):33096–110. doi: 10.18632/oncotarget.8861 (PMC5078078; doi:10.18632/oncotarget.8861)
Supplement: Supplementary file 1 [file oncotarget-07-33096-s001.pdf]

SUPPLEMENTARY FIGURES

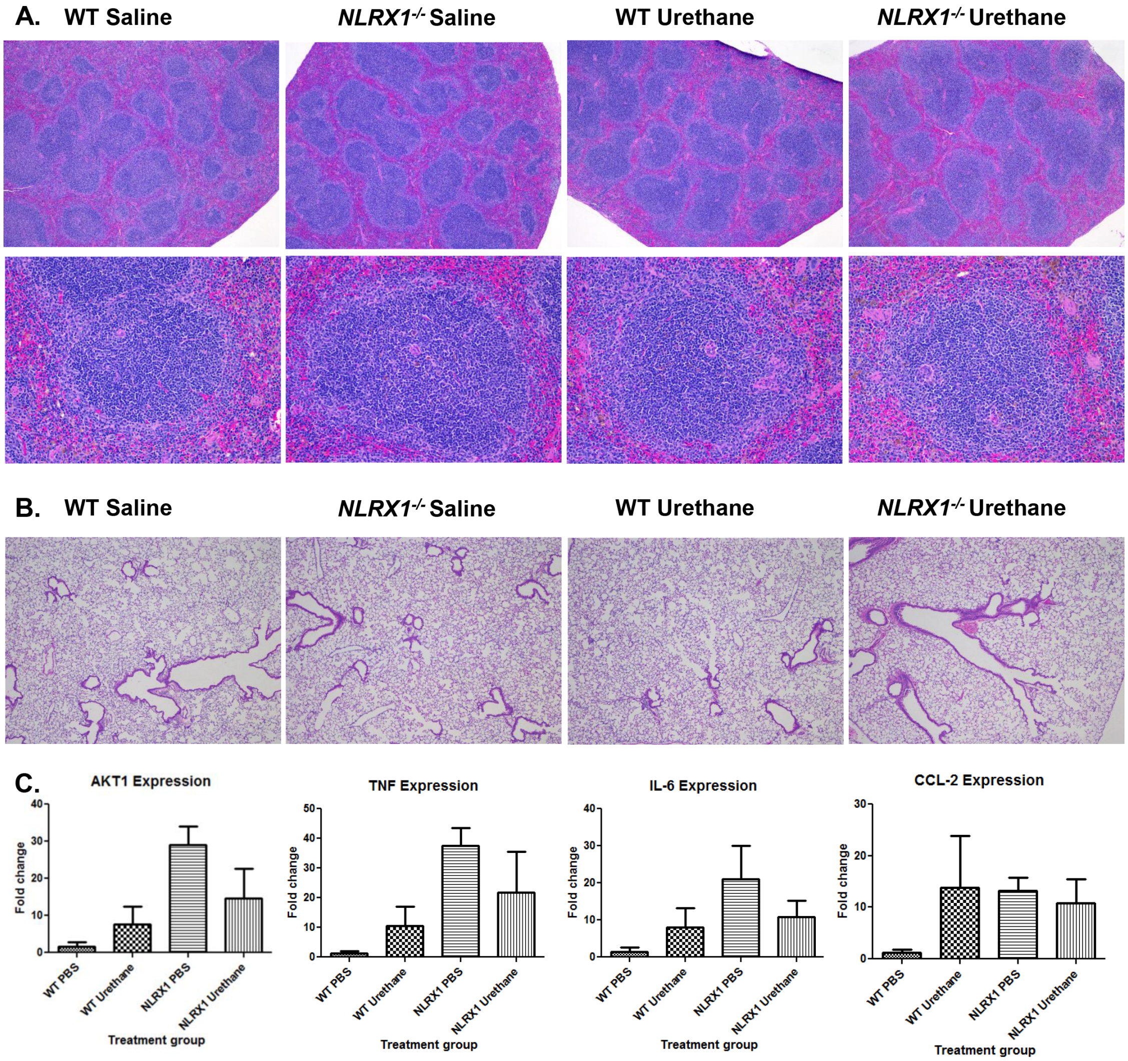

Supplementary Figure S1: Disease Progression and Changes in Gene Expression Identified at 14 Weeks are not Evident at 7 Weeks. A-B. Additional mouse studies evaluated at week 7 revealed no gross or histologic evidence of splenomegaly or tumorigenesis in the A) spleen or B) lung. **C.** No significant differences in gene expression of *Akt1*, *Tnf*, *Il-6*, *Nlrx1*, and *Ccl-2* were identified between the experimental and control groups. Analysis was based on the ΔΔCt method, where all data was standardized to the average gene expression for 18s and normalized to the respective untreated wild type spleens.

A.

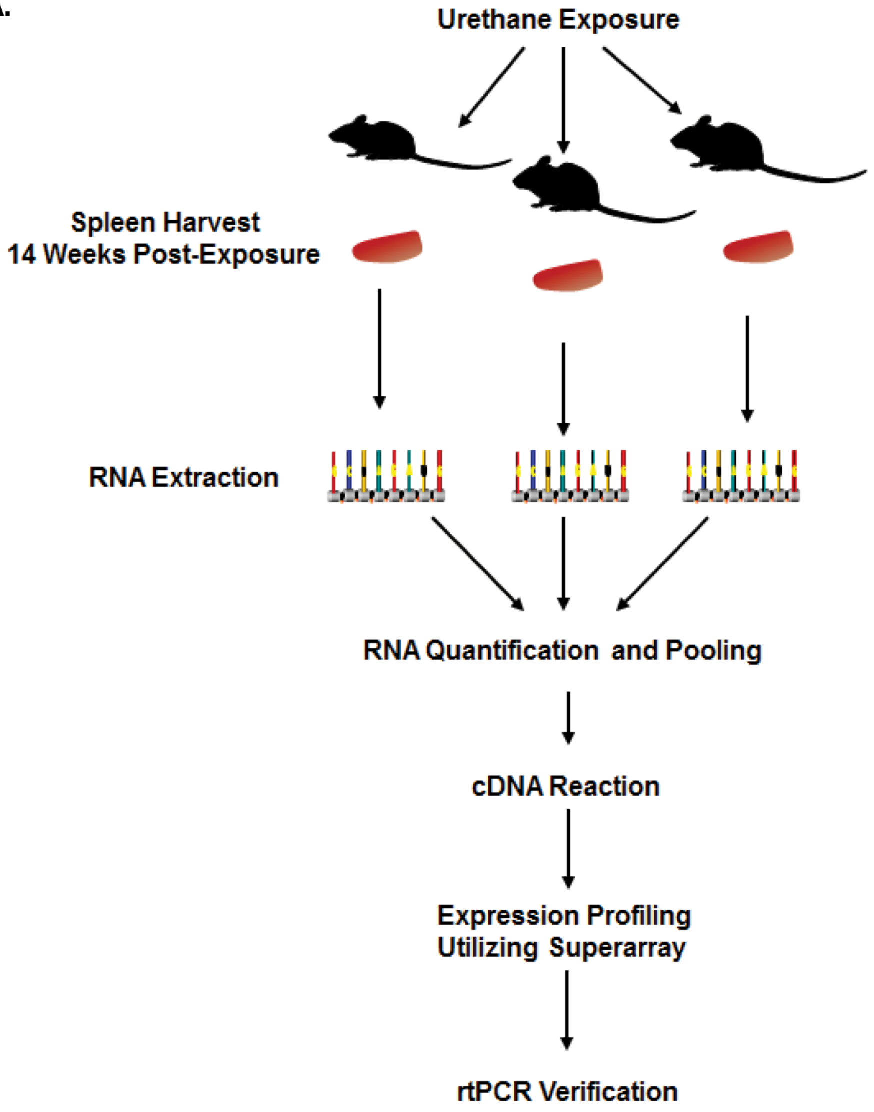

Supplementary Figure S2: Schematic Illustrating the Experimental Design for Expression Profiling. Multiple mice from each genotype and condition were exposed to urethane. Spleens were harvested 14 weeks following the initial urethane exposure. The total spleen RNA from each mouse was quantified and pooled in equal amounts. The pooled RNA was converted into cDNA and expression profiling was conducted using a panel of Superarrays (Qiagen). Expression data was verified by evaluation of gene expression changes of a subset of individual genes using rtPCR from both pooled and un-pooled samples.

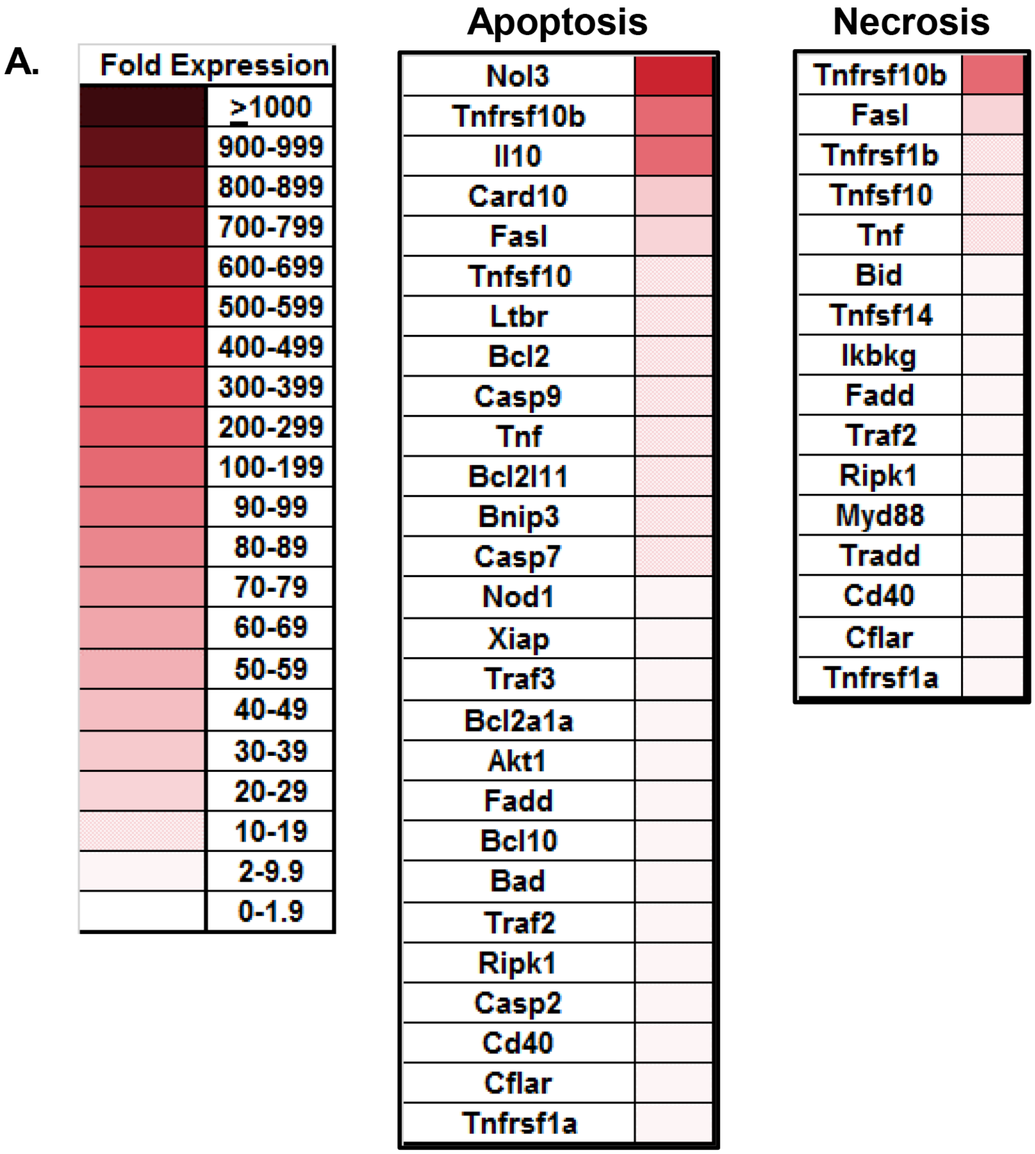

Supplementary Figure S3: Genes Associated with Both Apoptosis and Necrosis are Up-regulated During Tumorigenesis. Heatmap reflecting the change in gene expression of genes associated with cell death segregated into those associated with apoptosis versus necrosis. These genes were identified as being significantly up-regulated in the spleen following urethane treatment of *Nlrx1*$^{-/-}$ mice compared to the urethane treated wild type animals. Analysis was based on the ΔΔCt method, where all data was standardized to the average gene expression for a panel of 8 housekeeping genes and normalized to the respective untreated *Nlrx1*$^{-/-}$ and untreated wild type spleens. Greater than a 2-fold change in gene expression was considered significant. Three - five randomly selected spleens from each genotype and treatment group were selected and pooled for profiling studies.
